# Expression of the *Thermobifida fusca* xylanase Xyn11A in *Pichia pastoris* and its characterization

**DOI:** 10.1186/s12896-015-0135-y

**Published:** 2015-03-18

**Authors:** Longmei Zhao, Jiang Geng, Yaoqi Guo, Xiudong Liao, Xuhui Liu, Rujuan Wu, Zhaojun Zheng, Rijun Zhang

**Affiliations:** Laboratory of Feed Biotechnology, State Key Laboratory of Animal Nutrition, College of Animal Science and Technology, China Agricultural University, Beijing, 100193 China

**Keywords:** *Thermobifida fusca* Xyn11A, *Pichia pastoris*, Expression, C-terminal His-tag, Characterization, Glycosylation

## Abstract

**Background:**

Xylan is a major component of plant cells and the most abundant hemicellulose. Xylanases degrade xylan into monomers by randomly cleaving β-1,4-glycosidic bonds in the xylan backbone, and have widespread potential applications in various industries. The purpose of our study was to clone and express the endoxylanase gene *xynA* of *Thermobifida fusca* YX in its native form and with a C-terminal histidine (His) tag in *Pichia pastoris* X-33. We analyzed and compared these two forms of the protein and examined their potential applications in various industries.

**Results:**

The *xynA* gene from *T. fusca* YX was successfully cloned and expressed using *P. pastoris* X-33. We produced a recombinant native form of the protein (rXyn11A) and a C-terminal His-tagged form of the desired protein (rXyn11A-(His)_6_). The specific activities of rXyn11A and rXyn11A-(His)_6_ in culture supernatants approached 149.4 and 133.4 U/mg, respectively. These activities were approximately 4- and 3.5-fold higher than those for the non-recombinant wild-type Xyn11A (29.3 U/mg). Following purification, the specific activities of rXyn11A and rXyn11A-(His)_6_ were 557.35 and 515.84 U/mg, respectively. The specific activity of rXyn11A was 8% higher than that of rXyn11A-(His)_6_. Both recombinant xylanases were optimally active at 80°C and pH 8.0, and exhibited greater than 60% activity between pH 6–9 and 60–80°C. They exhibited similar pH stability, while rXyn11A exhibited better thermostability; N-glycosylation enhanced the thermostability of both recombinant xylanases. The products of beechwood xylan hydrolyzed by both xylanases included xylobiose, xylotriose, xylotetraose and xylopentaose.

**Conclusions:**

The C-terminal His tag had adverse effects when added to the Xyn11A protein. The thermostability of both recombinant xylanases was enhanced by N-glycosylation. Their stabilities at a high pH and temperature indicate their potential for application in various industries.

## Background

Xylan, the major component in plant cells and the most abundant hemicellulose, is composed of β-1,4-linked xylopyranosyl residues [1,2]. Recently, lignocellulose, the most plentiful renewable biomass produced from photosynthesis, has attracted worldwide attention as a raw material for bioconversion [3]. Degrading all components of lignocellulose requires the synergistic activity of a large variety of enzymes with different specificities, such as exo-1,4-β-glucanases, endo-1,4-β-glucanases, β-glucosidases, endo-xylanases, pectin methyl esterases, and laccase [4].

Among these, xylanases (β-1,4-Endoxylanases, EC 3.2.1.8) are the core enzymes responsible for the degradation of xylan into monomers through the random cleavage of β-1,4-glycosidic bonds in the xylan backbone [4,5]. Xylanases have widespread potential applications in the textile, feed, beverage, and biofuel industries [2,6-10]; moreover, they form a major group of industrial enzymes used in the paper and pulp industry. The release of lignin from paper pulp can be facilitated by the hydrolysis of xylan [2]. Xylanases can facilitate the hydrolysis of lignocellulosic substrates as xylanases ameliorate the “blocking effect” of xylan, one of the major mechanisms that limits the accessibility of cellulose to cellulase enzymes. Xylanases also interact synergistically with cellulases to improve cellulose accessibility by increasing fiber swelling and porosity [11].

According to amino acid sequences, structural folds, and catalytic mechanisms, xylanases are classified into glycoside hydrolases (GH) families 5, 7, 8, 10, 11, and 43 [12-14]. An industrially suitable xylanase should have certain specific properties; for example, high activity and stability at high temperatures (>70°C) or at high pH (>8.0) [15]. *Thermobifida fusca* is a thermophilic actinomycete and a major degrader of plant cell walls in heated organic materials such as compost piles and rotting hay [16]. The xylanase Xyn11A, from *T. fusca*, belongs to the G (11) family and contains a substrate-binding domain. Xyn11A has thermostable properties, unique among family G xylanases, that are especially useful for industrial purposes [17].

The yeast *Pichia pastoris* is an excellent and efficient system for the expression of secreted and intracellular proteins [18]. A number of xylanases from a variety of organisms have been cloned and expressed in *P. pastoris* [19-25]. Some xylanases have been cloned and expressed in the bacterium *Escherichia coli* [20,26-28]. A comparison of expression levels and the activities of products in *P. pastoris* and *E. coli* revealed that expression levels of active proteins in *P. pastoris* were much higher than those in *E. coli* [29]. Recombinant proteins produced in *P. pastoris* were very easily N-glycosylated *via* post-translational modification, although the properties of glycosylated proteins were not always distinct from unglycosylated proteins. The thermostability of recombinant proteins can be decreased or increased after glycosylation [29,30].

We selected the *xynA* gene encoding Xyn11A from *T. fusca* YX for cloning and expression in *P. pastoris*. To facilitate the purification of recombinant proteins, some of the proteins were fused to a polyhistidine tag (His) at the C-terminus [31]; however, recombinant proteins containing a His-tag can differ from their wild-type counterparts with respect to dimerization or oligomerization properties [32]. We therefore also attempted to express *xynA* in its native form in a *P. pastoris* expression system and compared the properties of the native form with those of the His-tagged form.

## Results and discussion

### Analysis of *xynA* gene from *T. fusca* genomic DNA

**Figure 1:**
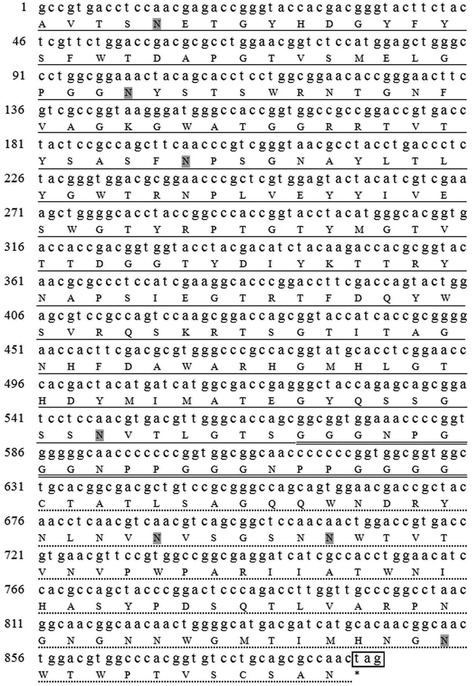
**Nucleotide sequence and corresponding amino acid sequence of the Xyn11A from** ***T. fusca*** **YX.** The catalytic domain of Xyn11A is underlined. The xylan binding domain of Xyn11A is indicated by a dotted underline. The Gly-Pro-rich linker region is double underlined. Seven putative N-glycosylation sites are highlighted in grey. The stop codon is boxed and marked by asterisk (*).

We amplified an 891-bp DNA fragment encoding the mature Xyn11A peptide (296 aa) by polymerase chain reaction (PCR) from the genomic DNA of *T. fusca* YX using primers (*xyA-f*, *xyA-r*, *xyA-his-r*). The fragment was then successfully cloned into the pMD19-T vector (TaKaRa, Dalian, China). According to the Xyn11A protein sequence (GenBank Accession Number U01242), the mature Xyn11A peptide comprises a 189-aa catalytic domain (CD), an 86-aa xylan binding domain (XBD), and a 21-aa Gly-Pro-rich region that connects the CD and XBD [17]. The nucleotide and corresponding aa sequences of Xyn11A are shown in Figure 1. Based on previous studies, there were seven possible N-glycosylation sites (Asn-X-Ser/Thr) [33], with very little O-linked glycosylation observed in *P. pastoris* [34]. Supposing that all of the possible N-glycosylations occurred and the average glycosylated side chain was Man_10_GlcNAc [34], then the molecular mass of the target protein would increase by about 12.9 kDa compared with the native wild-type Xyn11A (31.9 kDa).

### Expression of recombinant Xyn11A and Xyn11A-(His)_6_ in *P. pastoris*

The pPICZα-*xynA* and pPICZα-*xynA*-(His)_6_ expression plasmids were successfully generated as described in Methods. The coding sequences for Xyn11A and Xyn11A-(His)_6_ were cloned in frame with the α-factor signal sequence in the *P. pastoris-E. coli* shuttle vector pPICZα-A. After confirmation by sequencing, the recombinant plasmids were linearized using restriction endonuclease *Pme*I and electroporated into *P. pastoris* X-33. The pPICZα-A vector was used as a negative control and transformed into *P. pastoris* X-33. Transformants harboring pPICZα-*xynA* or pPICZα-*xynA*-(His)_6_ were grown on YPDS agar plates containing 100 μg/mL Zeocin and selected for high resistance to Zeocin (2000 μg/mL); successful transformants were obtained.

The pPICZα-*xynA* or pPICZα-*xynA*-(His)_6_ plasmids carrying the *xynA* gene were regulated by an alcohol oxidase gene promoter that could be induced by 0.5% (v/v) methanol. The maximum activities of recombinant Xyn11A (rXyn11A) and recombinant Xyn11A-(His)_6_ (rXyn11A-(His)_6_) in BMMY medium reached 1157.63 and 964.97 U/mL, respectively, after 96 h of cultivation. The specific activity of a crude preparation of rXyn11A (149.4 U/mg) was about 12% higher than that of rXyn11A-(His)_6_ (133.4 U/mg). The C-terminal His tag was located behind the XBD which was not essential for efficient hydrolysis of soluble xylan[17], therefore the C-terminal His tag affected the activity of rXyn11A-(His)_6_ through its dimerization or oligomerization properties [32].

**Figure 2:**
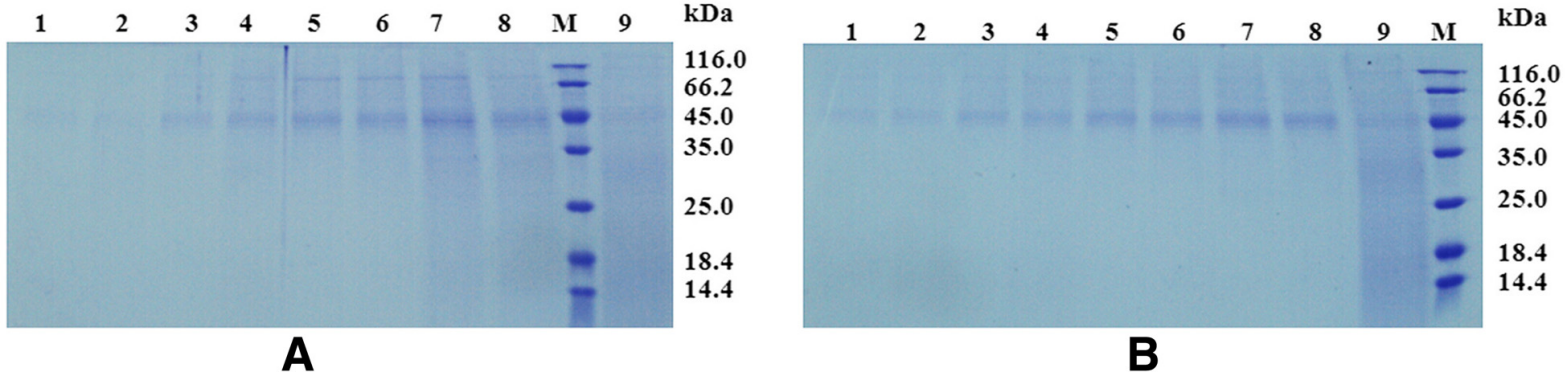
**Analysis of the accumulation of recombinant xylanases expressed in** ***P. pastoris X-33*** **.** Lane M: the molecular weight marker; **A**: SDS-PAGE of rXyn11A, Lane 1–8: supernatants of transformant harboring pPICZα-*xynA* obtained every 12 hours (12 h, 24 h, 36 h, 48 h, 60 h, 72 h, 84 h, 96 h), Lane 9: supernatant of transformant harboring pPICZα-A; **B**: SDS-PAGE of rXyn11A-(His)_6_, Lane 1–8: supernatants of transformant harboring pPICZα-*xynA*-(His)_6_ obtained every 12 hours (12 h, 24 h, 36 h, 48 h, 60 h, 72 h, 84 h, 96 h), Lane 9: supernatant of transformant harboring pPICZα-A. All samples containing 16 μL supernatant were loaded onto a 15% polyacrylamide gel.

**Figure 3:**
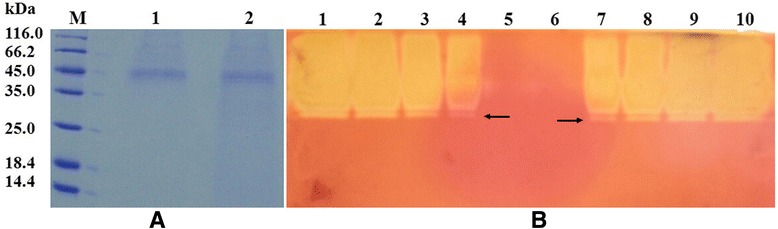
**Identification of the recombinant xylanases about 45 kDa.** Lane M: the molecular weight marker; **A**: SDS-PAGE of recombinant xylanases stained with Coomassie Brilliant Blue R-250 (samples containing 16 μL supernatant were loaded onto a 15% polyacrylamide gel), Lane 1: supernatant of transformant harboring pPICZα-*xynA*-(His)_6_, Lane 2: supernatant of transformant harboring pPICZα-*xynA*; **B**: SDS-PAGE activity staining of recombinant xylanases using Congo Red (samples were loaded onto a 15% polyacrylamide gel), Lane 1–4: supernatants of transformant harboring pPICZα-*xynA*-(His)_6_ with different loading volumes (15 μL, 10 μL, 5 μL, 2.5 μL), Lane 5–6: supernatant of transformant harboring pPICZα-A, Lane 7–10: supernatants of transformant harboring pPICZα-*xynA* with different loading volumes (2.5 μL, 5 μL, 10 μL, 15 μL).

Several protein bands corresponding to a molecular mass of about 45 kDa were observed as the major protein in supernatants from *P. pastoris*/pPICZα-*xynA* and *P. pastoris*/pPICZα-*xynA*-(His)_6_ cultures. This protein was not detected in the supernatant of *P. pastoris* cultures transformed with pPICZα-A (Figure 2). These 45-kDa proteins accumulated in supernatants obtained at different culture times (Figure 2). The molecular mass of native wild-type Xyn11A should be 31.9 kDa [17]; however, activity assays revealed that these 45-kDa proteins possessed xylanase activity (Figure 3), and thus we assign them as the recombinant xylanases with some glycosylated side chains. Xylanase activity was not detected in the supernatant of *P. pastoris* transformed with pPICZα-A. These results were consistent with those from previous studies, indicating the generation of several glycosylated proteins [15,34]. A separate protein band (Figure 3B, arrows) was thought to be the proteolytic degradation product of recombinant xylanase including the CD. The target proteins rXyn11A and rXyn11A-(His)_6_ constituted up to 63.7 and 62.3%, respectively, of all soluble proteins in supernatants as determined by Bandscan 5.0 software (Glyko, Novato, CA, USA).

### Purification of recombinant xylanases

**Figure 4:**
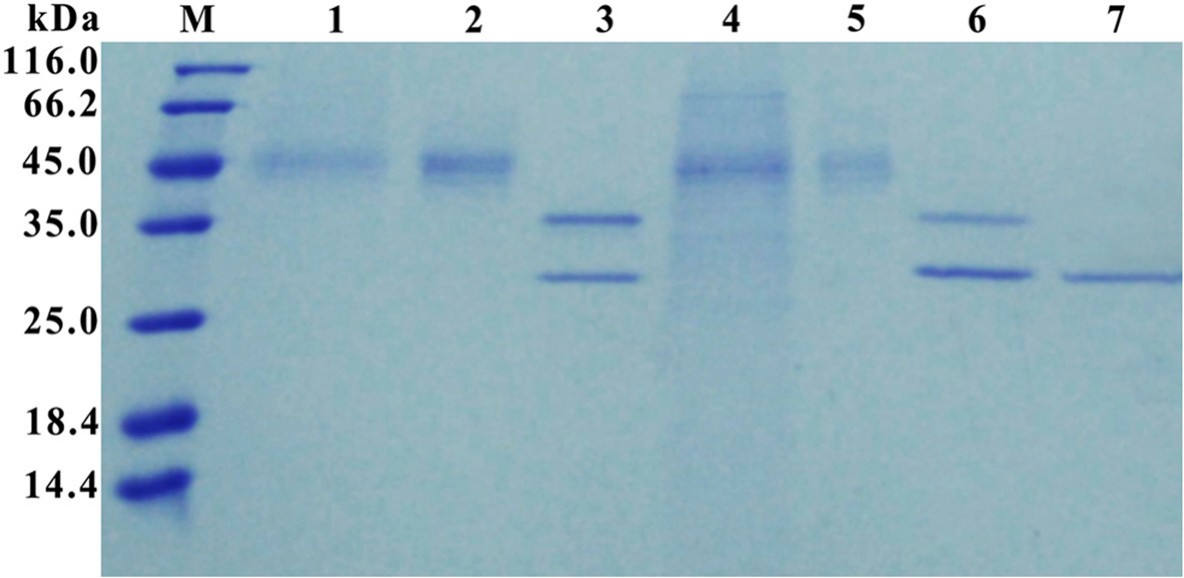
**SDS-PAGE analyses of the purification and deglycosylation of recombinant xylanases expressed in** ***P.pastoris X-33*** **.** Lane M: the molecular weight marker; Lane 1 and 4: supernatant of transformant harboring pPICZα-*xynA*-(His)_6_ and pPICZα-*xynA*, respectively; Lane 2 and 5: the purified rXyn11A-(His)_6_ and rXyn11A, respectively; Lane 3 and 6: the purified rXyn11A-(His)_6_ and rXyn11A deglycosylated with Endo H, respectively; Lane 7: Endo H.

Purification of the recombinant proteins was performed as described in Methods, and a summary is presented in Table 1. The rXyn11A and rXyn11A-(His)_6_ were purified 3.7-fold and 3.9-fold to homogeneity with recovery yields of 8.5% and 19.8%, respectively. The purification fold was not high because the recombinant xylanases constituted so much of the total protein in the supernatants (Figure 2). The specific activity of rXyn11A was 557.35 U/mg while that of rXyn11A-(His)_6_ was 515.84 U/mg. Purified fractions of rXyn11A and rXyn11A-(His)_6_ were subjected to 15% sodium dodecyl sulfate polyacrylamide gel electrophoresis (SDS-PAGE) and revealed several protein bands with molecular masses of about 45 kDa, which were distinct from the molecular mass of wild-type Xyn11A (31.9 kDa) and the calculated molecular mass of rXyn11A-(His)_6_ (32.7 kDa), respectively (Figure 4).

**Table 1:** **Summary of the purification of rXyn11A and rXyn11A-(His)**_**6**_

**Purification steps**	**Total activity (U)**	**Total protein (mg)**	**Specific activity (U · mg** ^**−1**^ **)**	**Purification (fold)**	**Yield (%)**
*P. pastoris* X-33/ pPICZα-*xynA*					
The supernatant	11576.3	77.5	149.4	1	100
Sephadex G-25	9205.5	36.1	254.9	1.7	79.5
Mono Q 5/50 GL	2462.3	6.3	390.2	2.6	21.3
Ultrafiltration	979.3	1.8	557.4	3.7	8.5
*P. pastoris* X-33/pPICZα-*xynA*-(His)_6_					
The supernatant	9649.7	72.4	133.4	1	100
Ni-NTA agarose	3057.0	15.8	193.6	1.5	31.7
Ultrafiltration	1908.6	3.7	515.8	3.9	19.8

Although rXyn11A-(His)_6_ could be purified to homogeneity with a simpler procedure than rXyn11A, the latter exhibited higher specific activity than the former, consistent with results from a previous study where it was shown that the recombinant native form of cellulase was more active and stable than a C-terminal His-tagged cellulase produced in *E. coli* [35].

### Characterization of the recombinant xylanases

**Figure 5:**
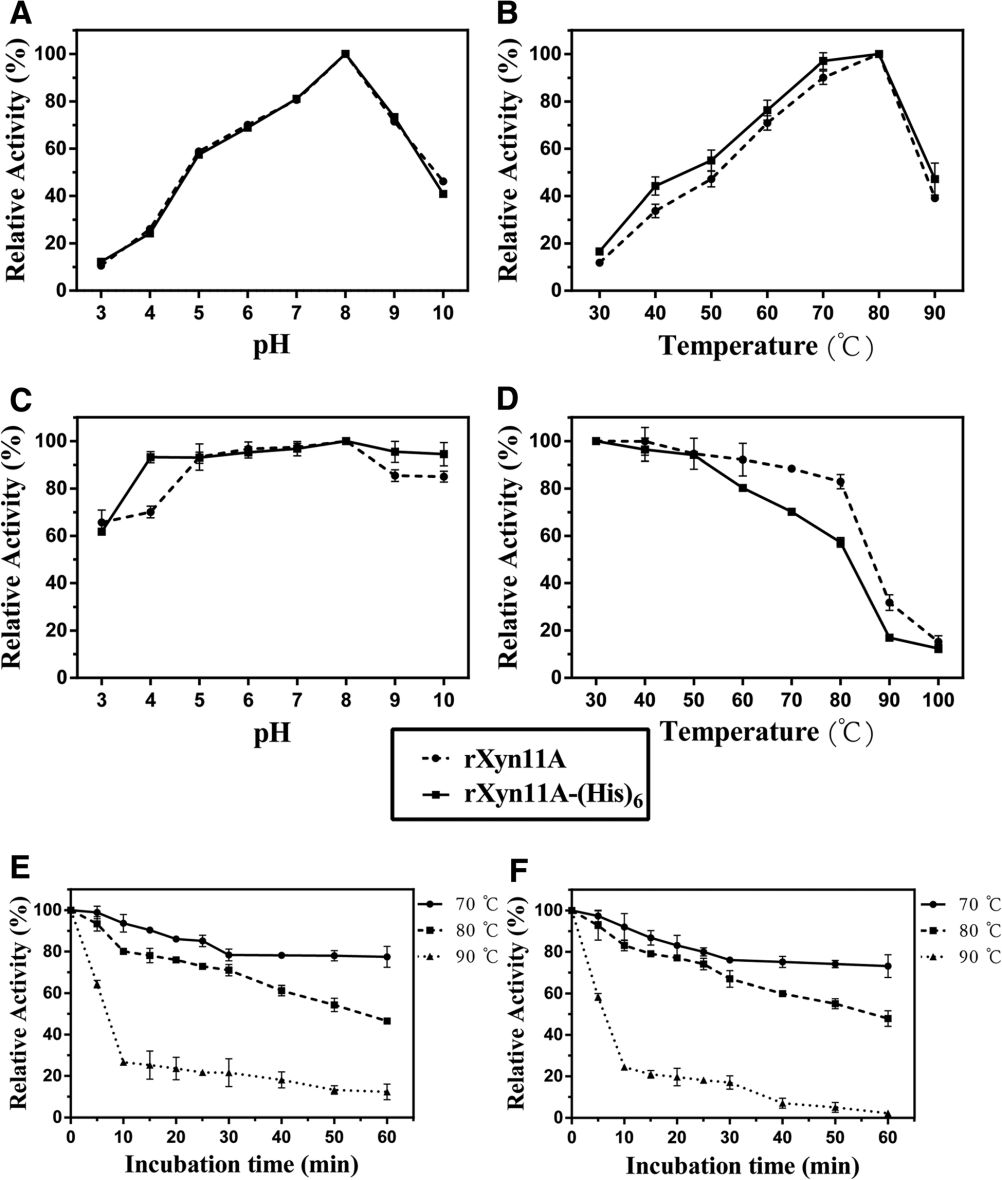
**Effect of pH and temperature on activity and stability of rXyn11A and rXyn11A-(His)**_**6**_ **. A**: Optimum pH; **B**: optimum temperature; **C**: pH stability; **D**: thermal stability; **E**: thermostability of rXyn11A (incubated at 70°C, 80°C, 90°C for different time); **F**: thermostability of rXyn11A-(His)_6_ (incubated at 70°C, 80°C, 90°C for different time).

Both rXyn11A and rXyn11A-(His)_6_ were optimally active at pH 8.0, and exhibited >60% activity between pH 6.0–9.0 (Figure 5A). Optimal activity was seen at 80°C, with >60% activity between 60 and 80°C (Figure 5B). Activities increased as the temperature rose, resulting in maximum activity at 80°C, and then decreased rapidly when the temperature increased above 80°C. The residual activities of both recombinant xylanases were >60% over a broad pH range (3.0–10.0), indicating their stability and that they would be of great practical use in various industrial applications (Figure 5C). The rXyn11A xylanase exhibited >80% residual activity between 30 and 80°C, while rXyn11A-(His)_6_ exhibited 57% residual activity at 80°C (Figure 5D). After incubation at 90°C for 30 min, rXyn11A and rXyn11A-(His)_6_ retained more than 10% of their initial activities (Figure 5D). We observed >20% residual activity for rXyn11A and rXyn11A-(His)_6_ after treatment at 90°C for 10 min (Figure 5E and F). Both of the recombinant xylanases showed better thermal stabilities than the native wild-type Xyn11A from *T. fusca* [17]. Compared with results in a previous study regarding the same xylanase that was not glycosylated in *P. pastoris* [36], rXyn11A and rXyn11A-(His)_6_ exhibited greater thermostability, possibly because of glycosylation of the recombinant xylanases.

### Analysis of deglycosylation

Analysis of rXyn11A and rXyn11A-(His)_6_ by SDS-PAGE revealed that their molecular weights (about 45 kDa) were increased by about 13 kDa compared with the native wild-type Xyn11A (31.9 kDa; Figure 4). Following treatment with endo-H to remove carbohydrate moieties, a protein band was observed at about 32 kDa by SDS-PAGE (Figure 4). These findings suggested that N-glycosylation of rXyn11A and rXyn11A-(His)_6_ accounted for about 13 kDa.

**Figure 6:**
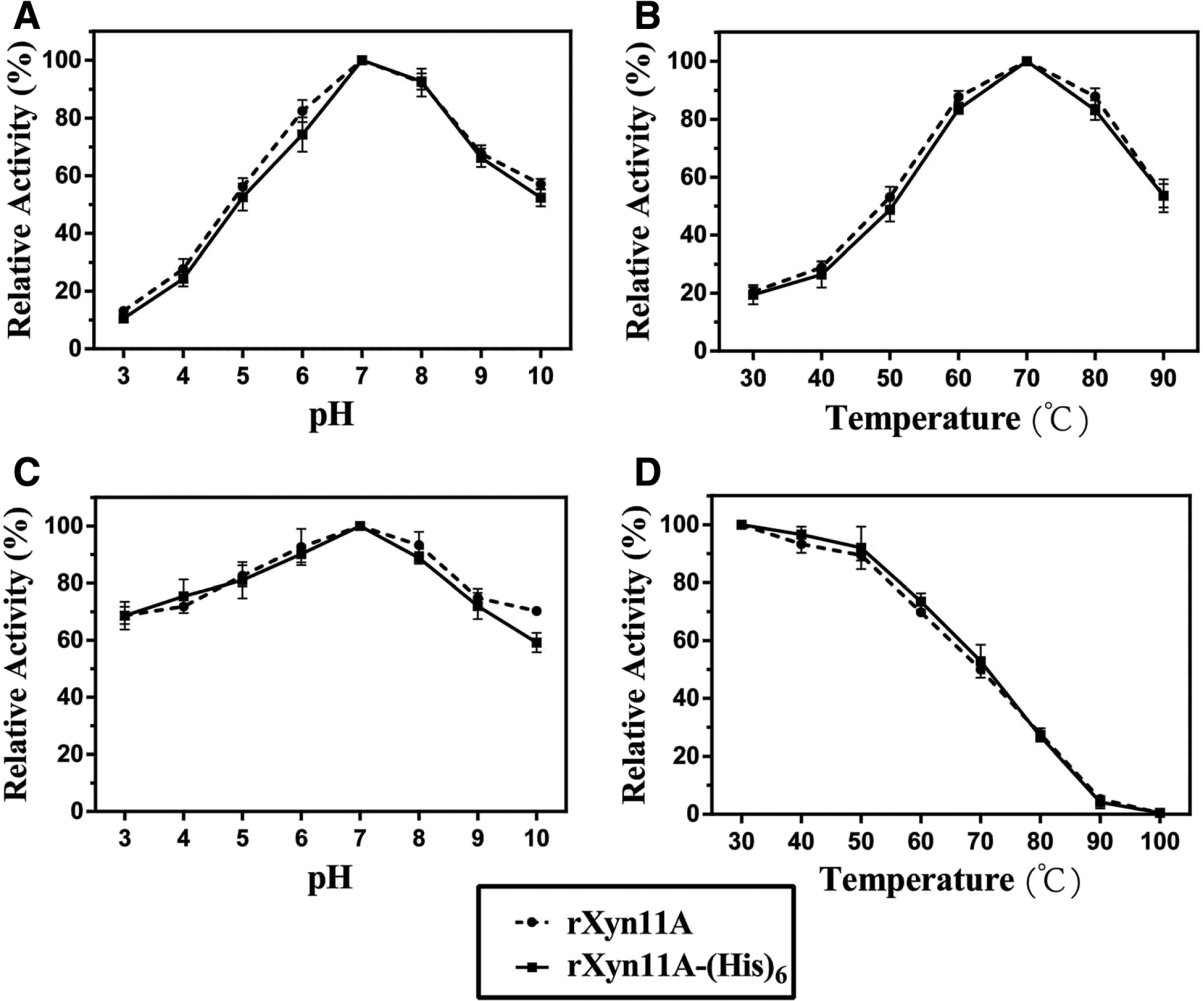
**Effect of pH and temperature on activity and stability of deglycosylated rXyn11A and rXyn11A-(His)**_**6**_ **. A**: Optimum pH; **B**: optimum temperature; **C**: pH stability; **D**: thermal stability.

**Figure 7:**
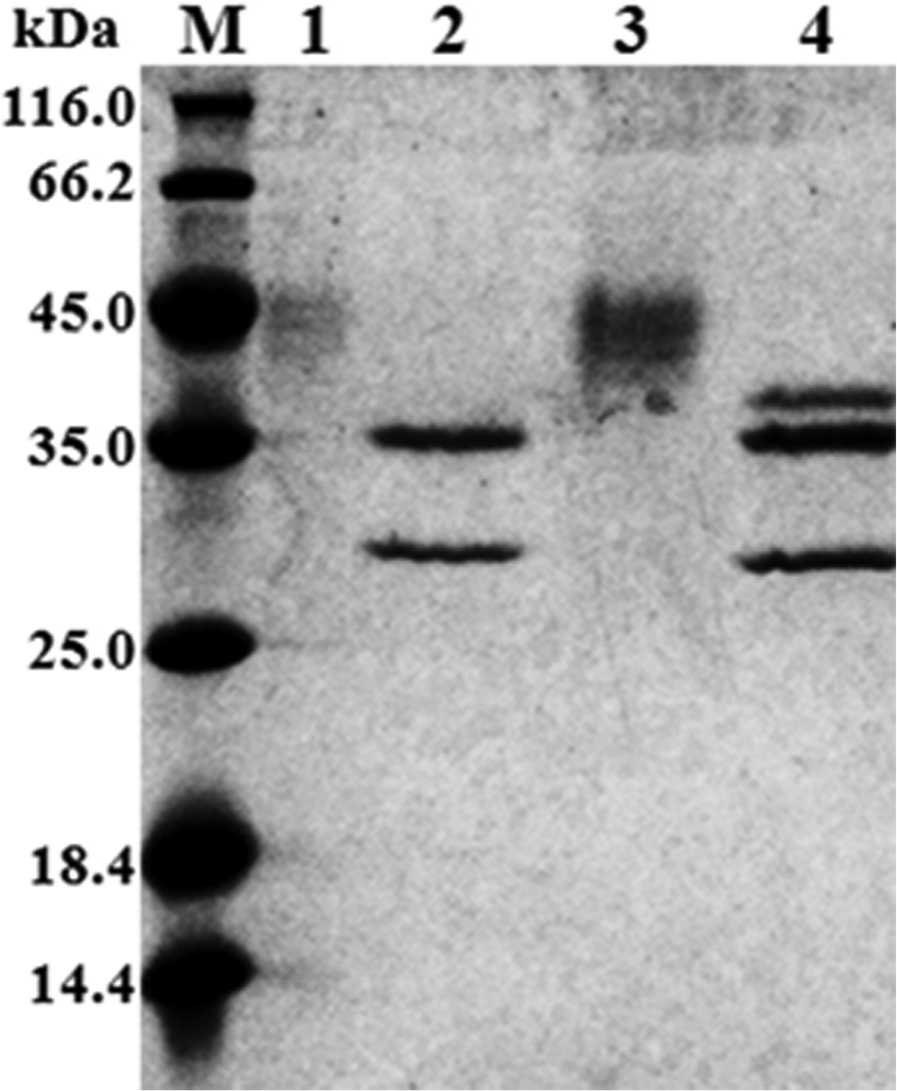
**SDS-PAGE analyses of deglycosylated rXyn11A and rXyn11A-(His)**_**6**_ **without denaturation.** Lane M: the molecular weight marker; Lane 1 and 3: the purified rXyn11A and rXyn11A-(His)_6_, respectively; Lane 2 and 4: the purified rXyn11A and rXyn11A-(His)_6_ deglycosylated with Endo H without denaturation, respectively.

Both the deglycosylated rXyn11A and rXyn11A-(His)_6_ showed optimal activities at 70°C and pH 7.0 (Figure 6A and B), which were decreased compared with the N-glycosylated recombinant xylanases (80°C and pH 8.0). The deglycosylated xylanases showed similar pH stability to the N-glycosylated recombinant xylanases (Figure 6C), but the thermostability was decreased. After incubation at 80°C and 90°C for 30 min, deglycosylated rXyn11A and rXyn11A-(His)_6_ retained less than 30% and 5% of their initial activities (Figure 6D), respectively. SDS-PAGE of deglycosylated rXyn11A and rXyn11A-(His)_6_ without denaturation showed that rXyn11A-(His)_6_ was not thoroughly deglycosylated (Figure 7), thus the change in thermostability of rXyn11A-(His)_6_ was less than that for rXyn11A. The C-terminal His tag did not have a major effect on the activity and stability of recombinant xylanase after deglycosylation (Figure 6).

The recombinant xylanases had higher optimum temperatures and exhibited better thermostability, possibly because of N-glycosylation. Our results corresponded with those in a previous study that showed that the N-glycosylation of recombinant enzymes expressed in *P. pastoris* enhanced their thermal stabilities [29,30,37].

**Figure 8:**
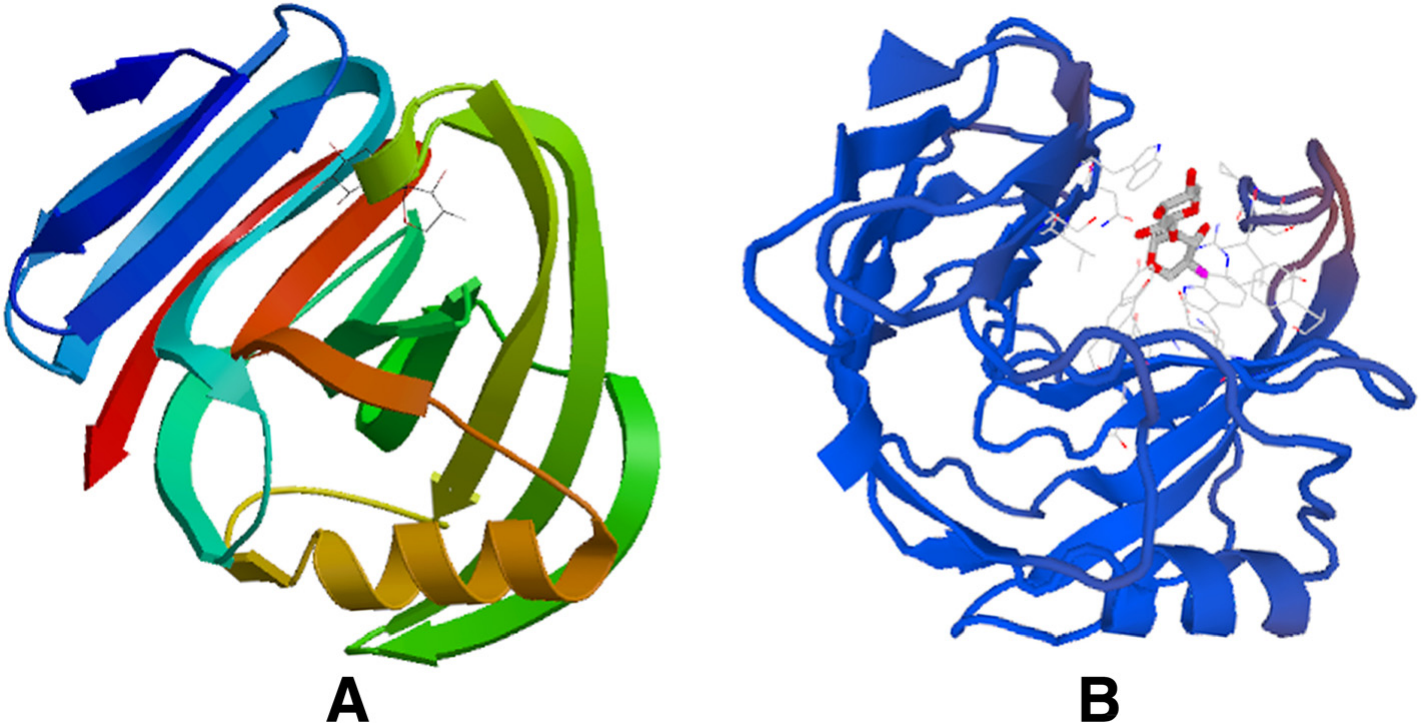
**Homology-modelling of Xyn11A. A**: Model of Xyn11A (the catalytic domain); **B**: Xyn11A model (the catalytic domain) showing the groove (housing the active site).

Based on the amino acid sequence of mature Xyn11A (Figure 1), there appear to be seven potential N-glycosylation sites (N-X-S/T: NET 5–7, NYS 34–36, NPS 66–68, NVT 183–185, NVS 230–232, NWT 236–238, NWT 285–287); these sites were predicted by NetNGlyc 1.0Server (http://www.cbs.dtu.dk/services/NetNGlyc/). Among these N-glycosylation sites, the first four were located within the CD and the last three sites were within the XBD. There were six sites (NET 5–7, NYS 34–36, NVT 183–185, NVS 230–232, NWT 236–238, NWT 285–287) with >54% probability of being glycosylated. A possible explanation for the higher thermostability caused by glycosylation may be that the long outer chains added during glycosylation can affect the folding or function of a foreign protein in *P. pastoris* [33]. In this study, the optimum pH was altered from 7.0 to 8.0 and the optimum temperature increased from 65°C to 80°C. These changes may be attributable to the four N-glycosylation sites located within the CD. To investigate the relationship between N-glycosylation and xylanase thermostability, we used Swiss Model to perform homology-modelling of Xyn11A (1–187 AA, the catalytic domain). There are one α-helix and several β-sheets in this domain (Figure 8A), and the β-sheets form a groove (Figure 8B) which houses the active site [38]. Several potential N-glycosylation sites were just included in the catalytic domain. Furthermore, it is believed that thermostable enzymes usually contain a large number of surface polar residues which generate more water-mediated networks surrounding the enzyme [38]. Therefore, the thermostability of rXyn11A and rXyn11A-(His)_6_ may be increased by N-glycosylation. The glycosylation of recombinant proteins alters some properties of the protein but not to a great extent [15,39]; in certain cases glycosylation can adversely affect thermostability in *P. pastoris* [29]. In a study examining the same xylanase, it was seen that when the linearized pPIC9K-tfx plasmid was transformed into *P. pastoris* GS115 after the xylanase gene was cloned into the vector, the expressed recombinant xylanase was not glycosylated and was the same size as the native xylanase [36]. These results showed that the difference between plasmids, as well as protein properties, may affect the glycosylation of recombinant proteins expressed in *P. pastoris*.

### Analysis of hydrolytic products

**Figure 9:**
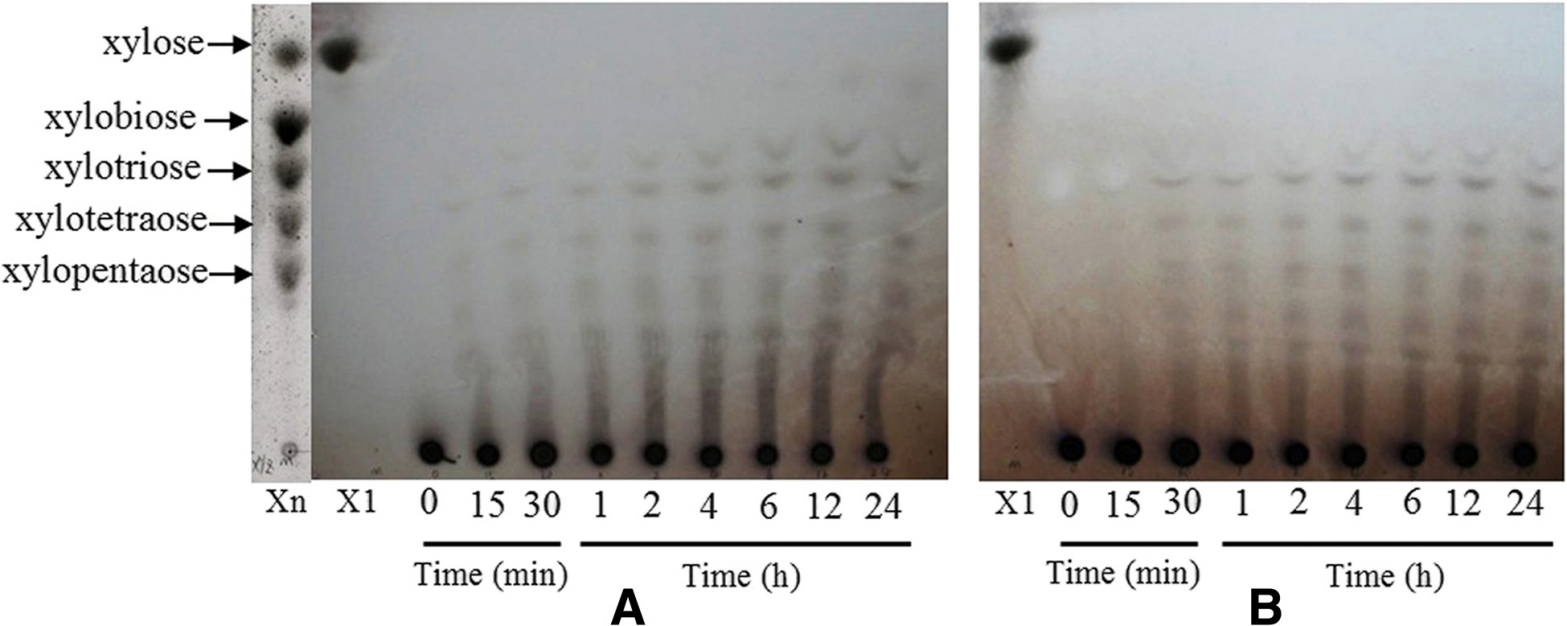
**Thin-layer chromatogram of hydrolytic products of beechwood xylan by recombinant xylanases. A**: Degradation of beechwood xylan by rXyn11A. **B**: Degradation of beechwood xylan by rXyn11A-(His)_6_.

The products of beechwood xylan hydrolyzed by rXyn11A and rXyn11A-(His)_6_ were analyzed using thin-layer chromatography (TLC) (Figure 9) and high-performance ion chromatography (HPIC) (Table 2). For TLC, a series of oligosaccharides with a degree of polymerization of two and above were observed on the silica gel plate. The main hydrolytic products were X2–X5, while many higher oligomers were still present in the digest. During hydrolysis, the concentrations of xylooligosaccharides increased for 4 h and then decreased as the xylooligosaccharides were hydrolyzed by the recombinant xylanases (Figure 9). The HPIC results revealed that rXyn11A is better at hydrolyzing beechwood xylan than rXyn11A-(His)_6_. These findings imply that the C-terminal His tag probably has adverse effects on xylanase activity. The C-terminal His tag may alter the structure of recombinant xylanases. The TLC and HPIC analyses showed that the main hydrolytic products were higher oligomers than xylose. Even after 24 h, there was little xylose released *via* hydrolysis. Therefore, our results indicate that rXyn11A and rXyn11A-(His)_6_ hydrolyzed xylan by cleaving the intramolecular glycosidic bonds of xylooligosaccharides, as they are both endoxylanases [17]. The distribution pattern of hydrolytic products was identical for rXyn11A and rXyn11A-(His)_6_.

**Table 2:** **Hydrolytic products concentration and percentage content of beechwood xylan by rXyn11A and rXyn11A-(His)**_**6**_

**Enzyme**	**Reaction time (h)**	**Hydrolytic products concentrations (μg/mL) and percentage content**				
		**Xylose**	**Xylobiose**	**Xylotriose**	**Xylotetraose**	**xylopentaose**
rXyn11A	6	12.0 (0.4%)	178.6 (6.0%)	630.8 (21.3%)	1007.9(34.1%)	1130.2(38.2%)
	12	16.9 (0.5%)	179.7 (5.4%)	661.5 (19.9%)	1113.1 (33.6%)	1347.6 (40.6%)
	24	24.1 (0.7%)	169.1 (5.2%)	649.6 (20.0%)	1152.6 (35.5%)	1255.3 (38.6%)
rXyn11A-(His)_6_	6	12.5 (0.8%)	69.8 (4.2%)	295.3 (18.0%)	593.1 (36.1%)	672.2 (40.9%)
	12	12.8 (0.7%)	90.2 (5.3%)	317.8 (18.5%)	589.0 (34.4%)	704.0 (41.1%)
	24	17.4 (1.1%)	85.5 (5.3%)	298.9 (18.5%)	552.5 (34.1%)	664.4 (41.0%)

## Conclusions

We have described the cloning and expression of a xylanase from *T. fusca*, and compared the characteristics of the recombinant wild-type xylanase, and the xylanase with a C-terminal His tag, expressed in *P. pastoris*. The characteristics of rXyn11A and rXyn11A-(His)_6_ were similar with respect to thermostability and pH tolerance. The activity of rXyn11A was higher than that of rXyn11A-(His)_6_, with both their activities higher than that of native Xyn11A. Therefore, we conclude that the C-terminal His tag had adverse effects on enzyme activity. N-glycosylation enhanced the thermostability of the recombinant xylanases, suggesting they could be applied to the degradation of cellulosic materials and in other industries.

## Methods

### Chemicals and enzymes

Restriction endonucleases, T4 DNA Ligase and Endoglycosidase H (Endo H) were purchased from New England Biolabs (Ipswich, MA, USA). Yeast extract and peptone were obtained from OXOID (Basingstoke, Hampshire, UK), yeast nitrogen base (YNB) without amino acids was purchased from BD (Sparks, MD, USA), biotin was obtained from Amersco (Solon, OH, USA), and Zeocin was purchased from Invitrogen (San Diego, CA, USA). Ni–NTA agarose and silica gel plates 60 F 254 was purchased from Merck (Darmstadt, Germany). Beechwood xylan was purchased from Sigma Chemical Company (St. Louis, MO, USA). Xylose (X1) was from Wako (Osaka, Japan). All other chemicals used were of reagent grade obtained from standard sources.

### Strains and plasmids

Genomic DNA from *T. fusca* YX used in this study was purchased from ATCC (catalog BAA-629D-5). For cloning, pMD19-T vector (TaKaRa, Dalian, China) and E. coli DH5α (Tiangen, Beijing, China) were used according to the manufacturers’ recommendation. *P. pastoris* X-33 and pPICZα-A vector from EasySelect Pichia Expression Kit were purchased from Invitrogen (San Diego, CA, USA).

### Construction of the plasmid for Xyn11A expression

In order to obtain the target gene *xynA* encoding the mature region of Xyn11A from *T. fusca* YX genome, three primers (*xyA-f*, *xyA-r*, *xyA-his-r*) were designed. The mature *xynA* gene was PCR amplified from genomic DNA of *T. fusca* YX with *xyA-f* (5′-CGGAATTCGCCGTGACCTCCAACGAGA-3′) as forward primer and *xyA-r* (5′-GCTCTAGATTACTA GTTGGCGCTGCAGGACA-3′) as reverse primer (*EcoR*I and *Xba*I restriction sites are denoted by the underline). For the PCR of His-tagged *xynA* gene, the reverse primer *xyA-his-r* (5′-GCTCTAGATTACTA ATGATGATGATGATGATGGTTGGCGCTGCAGGACA-3′) was used (*Xba*I restriction site and (His)_6_-tag are denoted by the underline). Both PCR amplifications were carried out in a Thermal cycler (Bio-Rad, Hercules, CA, USA) as the following conditions: an initial denaturation at 95°C for 5 min, 30 cycles of 95°C for 30 s, 55°C for 30 s and 72°C for 1 min, followed by one cycle of 72°C for 10 min. Afterwards, both PCR products were cloned into pMD19-T vector, yielding plasmids pMD19-*xynA* and pMD19-*xynA*-(His)_6_, respectively.

To obtain the secretive expression of rXyn11A and rXyn11A-(His)_6_, the plasmid pMD19-*xynA*/pMD19-*xynA*-(His)_6_ and *P. pastoris-E. coli* shuttle vector pPICZα-A were both digested with *EcoR*I and *Xba*I and mutually ligated. After being transformed into *E. coli* DH5α, transformants were selected at 37°C on the low salt LB agar plates (5 g/L yeast extract, 10 g/L tryptone, 5 g/L NaCl, 15 g/L agar, and adjusted pH to 7.5) containing 25 μg/mL Zeocin. The recombinant plasmids designated as pPICZα-*xynA* and pPICZα-*xynA*-(His)_6_ were obtained and sequenced by Invitrogen (Shanghai, China).

### Transformation of *P. pastoris* and screening of multi-copy transformants

Both the recombinant plasmids pPICα-*xynA* and pPICZα-*xynA*-(His)_6_ were linearized by restriction digestion with *Pme*I. Afterwards linearized DNA were transformed into *P. pastoris* X-33 by electroporation using MicroPulser (Bio-Rad, Hercules, CA, USA) under the PIC setting. Then the *P. pastoris* transformants were selected on the YPDS agar plates (10 g/L yeast extract, 20 g/L peptone, 20 g/L dextrose, 20 g/L agar and 1 M sorbitol) containing 100 μg/mL Zeocin at 30°C. After that, the transformants obtained were checked for the target gene integration using the extracted *P. pastoris* genomic DNA by PCR.

In order to get multi-copy transformants, the recombinant clones obtained were dotted onto the YPDS agar plates with higher Zeocin concentration (for example, 1000 μg/mL, 2000 μg/mL) and incubated at 30°C for about 2 days.

### Expression of recombinant xylanases in *P. pastoris*

For the expression of rXyn11A and rXyn11A-(His)_6_ in recombinant *P. pastoris*, a single colony was inoculated into 25 mL BMGY medium (10 g/L yeast extract, 20 g/L peptone, 100 mM potassium phosphate (pH 6.0), 13.4 g/L YNB, 4 × 10^−4^ g/L biotin, 1% (v/v) glycerol) in a 250 mL baffled flask and shaken (220 rpm) at 29°C for about 18 hours. Then the cells were harvested by centrifuging at 3000 × g for 5 min, and resuspended to an OD600 of 1.0 in BMMY medium (10 g/L yeast extract, 20 g/L peptone, 100 mM potassium phosphate (pH 6.0), 13.4 g/L YNB, 4 × 10^−4^ g/L biotin, 0.5% (v/v) methanol) about 100 mL in a 1 L baffled flask, shaken at 29°C for 4 days. To maintain induction, 100% methanol was added to the culture to a final concentration of 0.5% every 24 h during the induction phase.

### Purification of recombinant xylanases

For the purification of rXyn11A, the culture supernatant was collected by centrifuging at 12000 rpm for 5 min. The ÄKTA avant 25 chromatography system (GE Healthcare, Little Chalfont, UK) was used for the purification. The supernatant was loaded on a Sephadex G-25 (GE Healthcare, Little Chalfont, UK) column which was pre-equilibrated with 20 mM Tris–HCl buffer (pH 8.2). Binding protein was eluted by the same buffer, the eluted protein was pooled and loaded on an anion-exchange Mono Q column which was equilibrated with 20 mM Tris–HCl buffer (pH 8.2). Then the Mono Q column was eluted by 20 mM Tris–HCl buffer (pH 8.2) containing 0.9 M NaCl at 2 ml/min. Afterwards the purified protein was pooled and concentrated by the ultrafiltration with a 10 kDa molecular weight cut-off membrane in the Amicon Ultra-15 centrifugal filter unit (Merck Millipore, MA, USA). The concentrated protein was stored at 4°C.

For the purification of rXyn11A-(His)_6_, the harvested culture supernatant was filtrated with 0.22 μm Millex-GP Filter Unit (Merck Millipore, MA, USA) after centrifuging the culture at 12000 rpm for 5 min, and then the filtrated supernatant was directly applied to Ni-NTA column (Merck, Darmstadt, Germany) which was equilibrated with 50 mM PBS buffer (pH 7.4) at 1 mL/min. After the column was washed by 50 mM PBS buffer (pH 7.4) at 2 mL/min, the enzyme was eluted successively with 50 mM PBS buffer (pH 7.4) containing 50 mM and 100 mM imidazole at 2 ml/min until A280 of the effluent kept constant. The eluted enzyme was pooled and concentrated by the ultrafiltration with a 10 kDa molecular weight cut-off membrane in the Amicon Ultra-15 centrifugal filter unit (Merck Millipore, MA, USA). The purified enzyme was stored at 4°C for later use.

### Recombinant xylanase activity assays and protein determinations

The culture collected at 96 h was centrifuged and the xylanases activities of culture supernatants were measured [40] according to the increase in reducing sugar given by the dinitrosalicylic acid (DNS) method [41] using xylose as a standard. The reaction mixture containing 900 μL of 1% (w/v) beechwood xylan prepared in 0.2 M glycine-NaOH buffer (pH 8.0) and 100 μL of appropriately diluted xylanase enzyme solution was incubated at 80°C for 10 min. After the reaction was stopped by adding 1000 μL DNS solution (1% 3, 5-dinitrosalicyclic acid, 20% potassium sodium tartrate, 1% NaOH, 0.2% phenol, 0.05% Na_2_SO_3_) to the mixtures, the samples were vortexed and boiled for 10 min, followed by measuring the absorbance of 200 μL sample at 540 nm using iMark Microplate Reader (Bio-Rad, Hercules, CA, USA). One unit of xylanase activity was defined as the amount of xylanase required to catalyze the release of 1 μmol of xylose equivalent in 1 minute under the assay condition. All of the xylanase activity values presented were averages obtained from triplicate measurements. The protein concentrations of culture supernatant were measured by Bio-Rad Protein Assay kit (Hercules, CA, USA) in triplicate.

### SDS-PAGE and xylanase activity staining

SDS-PAGE was performed to detect the rXyn11A and rXyn11A-(His)_6_ with 5% stacking gel and 15% separating gel, and the protein bands were stained by Coomassie brilliant blue R-250.

Activity staining of recombinant xylanases was performed to display the visible xylanase activity bands on the gel [42] with slight modification. A 15% separating gel with 0.1% xylan in it was prepared for separating proteins. The protein samples prepared for the activity staining were heated for 10 min at 60°C in the presence of 2% SDS, but without 2-mercaptoethanol. After electrophoresis, the gel was immediately washed by five successive 30-min washes in 0.2 M cold glycine-NaOH buffer (pH 8.0) with first two washes contained 25% (v/v) isopropanol to remove the SDS. Then the gel was submersed in 0.2 M glycine-NaOH buffer (pH 8.0) and incubated at 80°C for 1 h. Subsequently, the gel was stained in 0.2% Congo Red for 30 min and destained with 1 M NaCl. Visible and clear activity bands against deep red background indicated the degradation of xylan.

### Deglycosylation of recombinant xylanases

Both the purified rXyn11A and purified rXyn11A-(His)_6_ were denatured by heating at 100°C for 10 min, and then the endoglycosidase H was added and the reaction mixtures were incubated at 37°C for 2 hour. And all manipulations were according to the instructions of manufacturer. Subsequently, SDS-PAGE was performed to detect the separation of reaction products.

For the activity assay of deglycosylated recombinant xylanases, the endo H digestion reactions were slightly adapted and conducted as follows, the reaction mixtures containing endo H and purified rXyn11A or rXyn11A-(His)_6_ were incubated at 37°C for 4 hour without denaturation process. Subsequently, SDS-PAGE was performed to detect the separation of reaction products.

### Characterization of recombinant xylanases in pH and temperature

To determine the optimal pH of rXyn11A and rXyn11A-(His)_6_ as well as deglycosylated rXyn11A and rXyn11A-(His)_6_, the xylanase activities were assayed in 0.2 M various buffers such as MacIlvaine’s buffer (pH 3.0-7.0) and glycine-NaOH buffer (pH 8.0-10.0) according to the standard assay method. For the determination of xylanases stabilities at various pH values, enzymes were pre-incubated in different pH buffers for 60 min at 55°C. After cooling, the residual activities were measured as the standard method.

To measure the temperature optima of rXyn11A and rXyn11A-(His)_6_ as well as deglycosylated rXyn11A and rXyn11A-(His)_6_, the xylanase activities were determined among the temperature range from 30°C to 90°C by the standard assay method. For the determination of xylanases thermostability, enzymes were pre-incubated at different temperatures (30–100°C) in 0.2 M glycine-NaOH buffer (pH 8.0) for 30 min, and the remained activities were measured as the standard method after cooling.

The thermostability of rXyn11A and rXyn11A-(His)_6_ was further investigated under different incubated time at 70, 80, 90°C. Aliquots were withdrawn at different time intervals (0 min, 5 min, 10 min, 15 min, 20 min, 25 min, 30 min, 40 min, 50 min, 60 min). After cooling, the remnant activities were assayed according to the standard method.

### Structural modelling of Xyn11A

The homology-modelling of Xyn11A was performed by Swiss-Model (http://swissmodel.expasy.org/) using the template (PDB entry 3zse), and the amino acid sequence identity was 99.47% [43,44].

### Analysis of hydrolysis of beechwood xylan by the recombinant xylanases

To analyze the hydrolytic products of beechwood xylan, 1.0% beechwood xylan in 2 ml of 0.2 M glycine-NaOH buffer was mixed with 10 μL of rXyn11A and 10 μL of rXyn11A-(His)_6_, respectively. The reaction mixtures were incubated at 80°C for 24 h. The aliquots at different time intervals were withdrawn and analyzed by both thin-layer chromatography (TLC) and high-performance ion chromatography (HPIC). For analysis of TLC, 10 μL of the samples were spotted on the silica gel plates 60 F 254 (Merck, Darmstadt, Germany). A mixture containing xylose (X1), xylobiose (X2), xylotriose (X3), xylotetraose (X4) and xylopentaose (X5) dissolved in water was used as the standard. Chromatography was developed in butanol-acetic acid-water (2:1:1, v/v) solvent system. After heating at 130°C for a couple of minutes in an oven, the plate was colorated by spraying with methanol-sulfuric acid mixture (95:5, v/v) and heated in an oven at 130°C for 5 min. The manipulations were modified from the method [45]. For analysis of HPIC, the samples at different time intervals (6 h, 12 h, 24 h) and standard xylooligosaccharides were analyzed with CarboPac^TM^ PA200 anion-exchange column (3 mm × 250 mm; Dionex, CA, USA), pure water as mobile phase (0.45 mL/min), and injection volumes were 10 μL. Sugar peaks were screened by ICS 5000 Electrochemical Detector (Dionex, CA, USA).

## Abbreviations

rXyn11A: Recombinant Xyn11A
rXyn11A-(His)6: Recombinant Xyn11A with C-terminal His-tagged
CD: Catalytic domain
XBD: Xylan binding domain
X1: Xylose
X2: Xylobiose
X3: Xylotriose
X4: Xylotetraose
X5: Xylopentaose
PCR: Polymerase chain reaction
YPDS: Yeast extract peptone dextrose medium with 1 M sorbitol
BMGY: Buffered glycerol-complex medium
BMMY: Buffered methanol-complex medium
PBS: Phosphate buffer saline
SDS–PAGE: Sodium dodecyl sulphate polyacrylamide gel electrophoresis
TLC: Thin-layer chromatography
HPIC: High-performance ion chromatography
